# Optimizing Mycoprotein Production by *Aspergillus oryzae* Using Soy Whey as a Substrate

**DOI:** 10.3390/jof11050349

**Published:** 2025-05-01

**Authors:** Ferren Pratama, Richelle Tirta Rahardja, Angellique Regina Rachmadi, Wildan Qoharisma Salam, Katherine Kho, Aivyanca Adelie, Putu Virgina Partha Devanthi

**Affiliations:** 1 Department of Biotechnology, School of Life Sciences, Indonesia International Institute for Life Sciences, Pulomas Barat Kavling 88, Jakarta 13210, Indonesia; ferren.pratama@alumni.i3l.ac.id (F.P.); richelle.rahardja@alumni.i3l.ac.id (R.T.R.); angellique.rachmadi@alumni.i3l.ac.id (A.R.R.); wildan.salam@i3l.ac.id (W.Q.S.); katherine.k@i3l.ac.id (K.K.); aivyanca.adelie@alumni.i3l.ac.id (A.A.); 2 School of Science, Constructor University, 28759 Bremen, Germany

**Keywords:** mycoprotein, edible fungi, *Aspergillus oryzae*, soy whey, tofu wastewater, alternative substrate, waste valorization, circular economy

## Abstract

Soy whey, a by-product of soy processing, has shown promise as a substrate for mycoprotein production using *Aspergillus oryzae*. However, the low biomass concentration obtained necessitates optimization of cultivation conditions to enhance total protein production. In this study, we optimized substrate concentration (50%, 75%, and 100%), initial pH (unadjusted, 4, 5, and 6), and supplementation with 8 g/L ammonium sulfate, minerals (0.75 g/L MgSO_4_·7H_2_O, 1 g/L CaCl_2_·H_2_O and 3.5 g/L KH_2_PO_4_), or their combination to maximize biomass production. The results showed that adjusting the initial pH to 5 and adding ammonium sulfate and minerals increased biomass concentration by 169% from 1.82 g/L to 4.9 g/L in 100% soy whey. This optimized condition also slightly improved the protein content of the biomass from 53% *w*/*w* to 55.93% *w*/*w*. Additionally, cultivating *A. oryzae* under these optimized conditions significantly reduced soy whey’s chemical oxygen demand from 8100 mg/L to 3267 mg/L, highlighting bioremediation potential. These findings suggest that the optimized conditions enhance the productivity of mycoprotein and also contribute to the sustainable management of soy whey waste, providing a combined benefit of nutrient recovery and wastewater treatment.

## 1. Introduction

Proteins are an essential part of the human diet, necessary for various bodily functions, including maintaining muscular and neurological activities [1,2]. However, high-quality protein sources are often obtained from animals, which can be expensive and, therefore, unaffordable for many people in marginalized communities [3]. This limited access can lead to insufficient protein intake, resulting in health problems such as kwashiorkor and stunting in children, which are prevalent in many developing and poor countries [3,4,5]. Moreover, current protein production that relies heavily on livestock has faced criticism due to sustainability concerns and environmental issues [6,7,8]. Sustainable and safe livestock production would require more investment, and increasing its production to keep up with the demand would be costly [6,9,10]. These challenges highlight the need for more accessible, affordable, and sustainable protein sources to address global nutritional needs.

A popular alternative to animal-based proteins is plant-based protein, which requires less resources, generates less waste, and overall generates ~50% less greenhouse gas emissions [11,12]. However, plant-based proteins are less digestible and contain fewer essential amino acids, vitamins, and minerals [13,14]. Insects are another alternative known for their high protein content but have poor consumer acceptance and are at risk of carrying pathogens [14]. Microbe-derived proteins are primarily derived from algae and fungi. Algae are highly digestible and consist of high protein content along with other micronutrients, but they have unsavory organoleptic properties [14,15,16]. Fungal proteins, particularly mycoproteins, contain more digestible protein and essential amino acids compared to plant-based proteins while also possessing higher amounts of fiber, minerals, and vitamins compared to animal-based proteins [6,17,18,19]. Current commercial mycoprotein production is dominated by Quorn^TM^, which utilizes *Fusarium venenatum* [18], while Fy protein uses *Fusarium flavolapis*, and Rhiza protein is derived from *Neurospora crassa* [20]. However, these commercial products also typically utilize expensive glucose syrup as the main carbon source for fungal growth [20,21]. Lastly, the cost of mycoprotein production is found to be less competitive per kg of protein compared to beef and chicken but can be improved by utilizing edible food by-products to allow more accessibility to lower-income communities [22,23].

Indeed, various studies have been performed utilizing other fungi in alternative substrates, such as *Aspergillus oryzae*, which is known for its adaptability while producing highly proteinaceous biomass (%) from paper, fish, and pea-processing by-products [24,25,26]. Notably, *A. oryzae* was found to produce the highest protein yield (0.26 g/g DW substrate) in pea-processing by-product compared to other fungal strains, including *F. venenatum*, *Neurospora intermedia*, *Rhizopus oryzae*, and *Monascus purpureus* [24]. Furthermore, *A. oryzae* has a long history of safe use in the fermentation of soy products, such as soy sauce and *miso* [27], and in our previous study, *A. oryzae* was capable of growth on soy-based by-products, including okara (soy pulp) and soy whey (tofu wastewater) [28]. Tofu is a widely consumed soy-based food, especially in Asia, which generates an abundance of wastewater, causing pollution due to a lack of feasible treatment [29,30]. However, the waste still consists of nutrients, which can be used for microbial cultivation [28,30,31].

Our previous study demonstrated that *A. oryzae* grown in soy whey, compared to okara, gave the highest dried biomass yield per dried substrate (408 mg/g DW substrate) and protein concentration (53% *w*/*w*). However, the dried biomass concentration from soy whey was significantly lower (0.87 g DW/L) compared to other studies, such as oat flour (6 g DW/L) and starch wastewater (12 g DW/L) [28,32,33]. While the high biomass yield indicates more efficient substrate utilization in soy whey compared to okara, the low biomass concentration represents a significant limitation for large-scale production, as it means less total biomass (and thus protein) is produced per batch. This is likely due to the unoptimized cultivation conditions, including the low initial pH (3.7), excessive cultivation time (7 days), and low substrate concentration (50%). To elaborate, soy whey is found to only contain 1% of carbohydrates (stachyose, raffinose, sucrose, glucose, and fructose), ~0.6% of nitrogen, and ~0.4% of ash [30,34], which was not sufficient for optimal biomass production. Furthermore, the nitrogen present in soy whey may be difficult to utilize, as previously, it was found that only ~40% of the total proteins were used. Yang et al. [35] also reported finding <0.05% of potassium, magnesium, and calcium each in soy whey, although these minerals have been found to support the growth of *A. oryzae* [36]. Therefore, supplementation of these nutrients may be necessary to improve biomass production by *A. oryzae*. Altogether, the combined factors in the unoptimized condition in soy whey substrate potentially further exacerbated mycelial biomass production. Hence, the aim of this study is to improve biomass production by *A. oryzae* by optimizing the substrate concentration, incubation time, and initial pH, along with supplementation of nitrogen and/or mineral sources.

## 2. Materials and Methods

### 2.1. Media and Reagents

Potato dextrose agar (PDA) (Merck, Darmstadt, Germany) was used for the maintenance of *A. oryzae*. Tofu or soy whey was provided by a small-scale tofu producer based in Bekasi, Indonesia, and stored at −20 °C until use. NaOH, MgSO_4_·7H_2_O, CaCl_2_·H_2_O, and KH_2_PO_4_ were reagents used to investigate the optimal cultivation parameters and were obtained from Merck, Darmstadt, Germany. Ammonium sulfate was obtained from Pudak Scientific, Bandung, Indonesia, and sodium chloride was obtained from Himedia, Mumbai, India.

### 2.2. Microorganism Maintenance and Media Preparation

*A. oryzae* was obtained from the Indonesia International Institute for Life Sciences (i3L)’s culture collection. The culture was previously isolated from commercially available koji starter culture (GEM cultures, Lakewood, WA, USA). Working stock cultures of *A. oryzae* were maintained on PDA and stored at 4 °C. The inoculum used for mycoprotein production was freshly prepared by transferring a fragment of mycelium onto fresh PDA and then allowing it to incubate for 7 days at 30 °C. The fungal spore suspension was obtained by flooding the PDA cultures with 15 mL of 0.85% NaCl solution, followed by gently scraping off the spores using a sterile 100 μL micropipette tip. The spore concentration was then adjusted to ~10^6^ spores/mL and immediately used.

### 2.3. Optimization of Cultivation Conditions for Mycelial Biomass in Soy Whey Media

**Figure 1:**
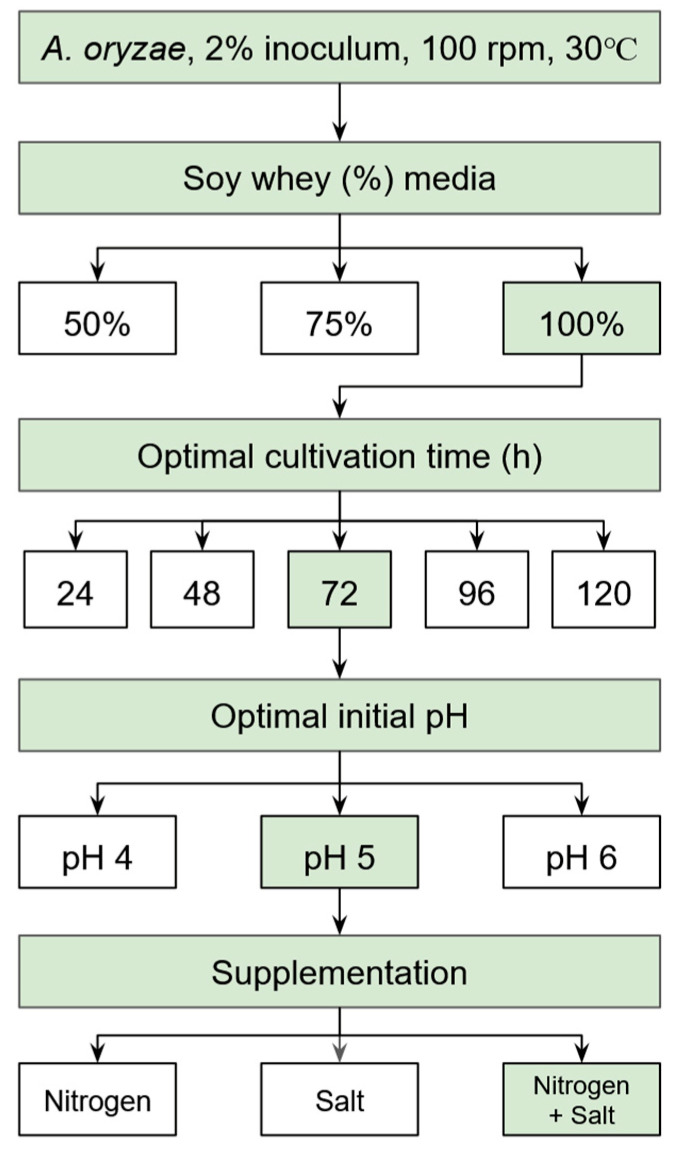
Flowchart of experimental design to determine optimal conditions for *A. oryzae* biomass production in soy whey media by investigating one factor at a time.

As the soy whey media in the previous study was able to support *A. oryzae*’s activity [28], investigations were performed to further improve biomass production (Figure 1). Cultivation factors, including substrate concentration (50%, 75%, and 100% *v*/*v*), cultivation time (24, 48, 72, 96, and 120 h), initial pH (4, 5, and 6), and nitrogen (8 g/L ammonium sulfate) and/or mineral (0.75 g/L MgSO_4_·7H_2_O, 1 g/L CaCl_2_·H_2_O and 3.5 g/L KH_2_PO_4_) supplementation, were optimized sequentially using the one-factor-at-a-time (OFAT) approach. In this method, each factor was optimized individually, and its optimal value was kept constant while optimizing the subsequent factor, as illustrated in Figure 1.

For each set of experiments, 49 mL of the prepared soy whey media was added into 250 mL Erlenmeyer flasks, autoclaved at 121 °C for 15 min, and stored at 4 °C until use. Then, 1 mL of prepared *A. oryzae* spore solution (~10^6^ spores/mL) was added into each flask, and the flasks were then incubated at 30 °C with agitation at 100 rpm. Samples were collected every 24 h until the end of cultivation, and the results are reported as the average of the triplicate samples.

### 2.4. Experimental Analysis

After cultivation, the samples were filtered using Whatman paper to separate the fungal biomass from the supernatant. The biomass was rinsed with distilled water twice and dried at 60 °C until a constant weight was achieved. Once the weight was stable, the biomass dry weight was measured, and the biomass concentration was calculated by dividing the total dry weight (g) by the total media volume (L). The fungal biomass was homogenized in distilled water using mortar and pestle as described by Khan et al. [37], and the protein content of the fungal biomass was measured using the bicinchoninic acid method according to the manufacturer’s instructions (Vazyme, Nanjing, China). The biomass and protein yield were calculated by dividing the obtained biomass or protein content (mg) by the amount of dried substrate (g) in the media.

Along with biomass characterization, the media and filtrate of the cultures were also characterized. The pH of the obtained supernatant was measured using a pH meter (OHAUS, Parsippany, NJ, USA), and the rest were collected and stored at −20 °C for further sugar and chemical oxygen demand (COD) analysis. The amount of sucrose, glucose, and fructose in the samples was measured using an enzyme kit (Megazyme, Bray, Ireland). The initial and final COD of the samples containing 100% soy whey without any adjustments and with adjustments (pH 5, supplemented with ammonium sulfate and minerals) were measured using a COD kit (HACH, Loveland, CO, USA). Samples were diluted using deionized water to fit within the range of detection of the analysis methods. The samples were then heated at 150 °C for 2 h in a DRB-200 digester, and once the samples were cooled to room temperature, the absorbance was measured using a DR-3900 spectrophotometer. All equipment used for COD analysis was obtained from HACH, Loveland, CO, USA.

### 2.5. Statistical Analysis

The data collected were analyzed using GraphPad Prism version 10.4.0. (GraphPad Software, Inc., San Diego, CA, USA). In particular, a one-way ANOVA followed by Tukey’s HSD test was performed to investigate the differences in final biomass concentration, biomass yield, protein content, protein yield, and COD activity. The effect of a specific condition was considered statistically significant if the *p*-value was less than the selected significance level (*p* < 0.05).

## 3. Results and Discussion

### 3.1. Optimized Cultivation Conditions for A. oryzae Biomass Production in Soy Whey

In our previous study, we demonstrated the ability of *A. oryzae* to produce biomass with a protein concentration of 53% *w*/*w* when cultivated in 50% soy whey for 7 days [28]. These findings confirmed the potential of soy whey as a viable substrate for supporting the growth of protein-rich biomass. However, despite the high protein content and biomass yield, the biomass concentration was relatively lower (0.87 g DW/L) than the values reported in other studies. It is worth noting that the cultivation was carried out under unoptimized conditions, as the primary goal was to explore the feasibility of soy whey as a growth medium rather than to maximize biomass production. Building on the previous findings, the current study aims to optimize cultivation conditions to obtain a higher concentration of *A. oryzae* biomass and, consequently, a higher protein yield from soy whey using the OFAT approach, similarly performed in other studies [38,39]. Key factors, including substrate concentration, cultivation time, initial pH, and the supplementation of nitrogen and minerals, were evaluated in a step-wise manner (Figure 1). The effects of each factor are presented and discussed in the following sections, following the order of the optimization steps.

#### 3.1.1. Effect of Substrate Concentration and Incubation Time

In our preliminary work, the feasibility of soy whey as a growth medium for *A. oryzae* was evaluated using 50% diluted soy whey [28]. While studies suggest that using diluted media can mitigate the potential inhibitory effects of excessive nutrient concentrations [40] or reduce the presence of potential trypsin inhibitors [41,42], such dilution may also result in nutrient levels that are too low to sufficiently support microbial growth [43]. However, *A. oryzae* was capable of growing in 50% soy whey, even under unoptimized conditions, and the growth may have been limited due to the low sugar content (~30 mg/L sucrose, 27 mg/L fructose, 1 mg/L glucose) [28]. Therefore, this experiment aims to investigate the effect of higher soy whey concentration on the metabolic activity and growth of *A. oryzae*.

**Figure 2:**
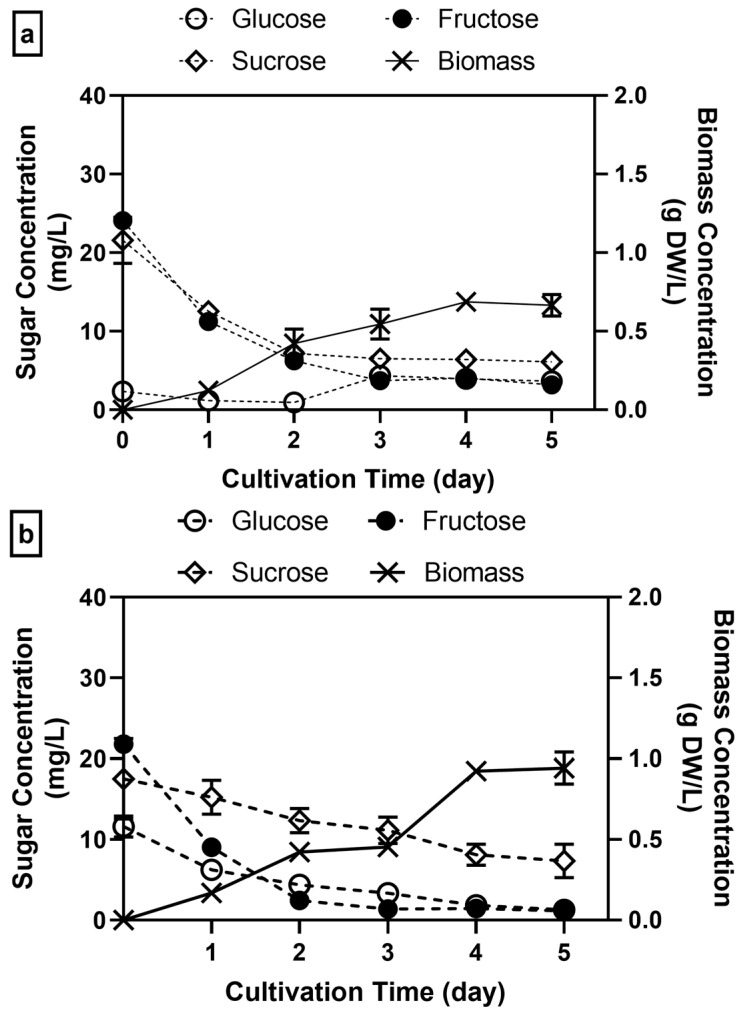

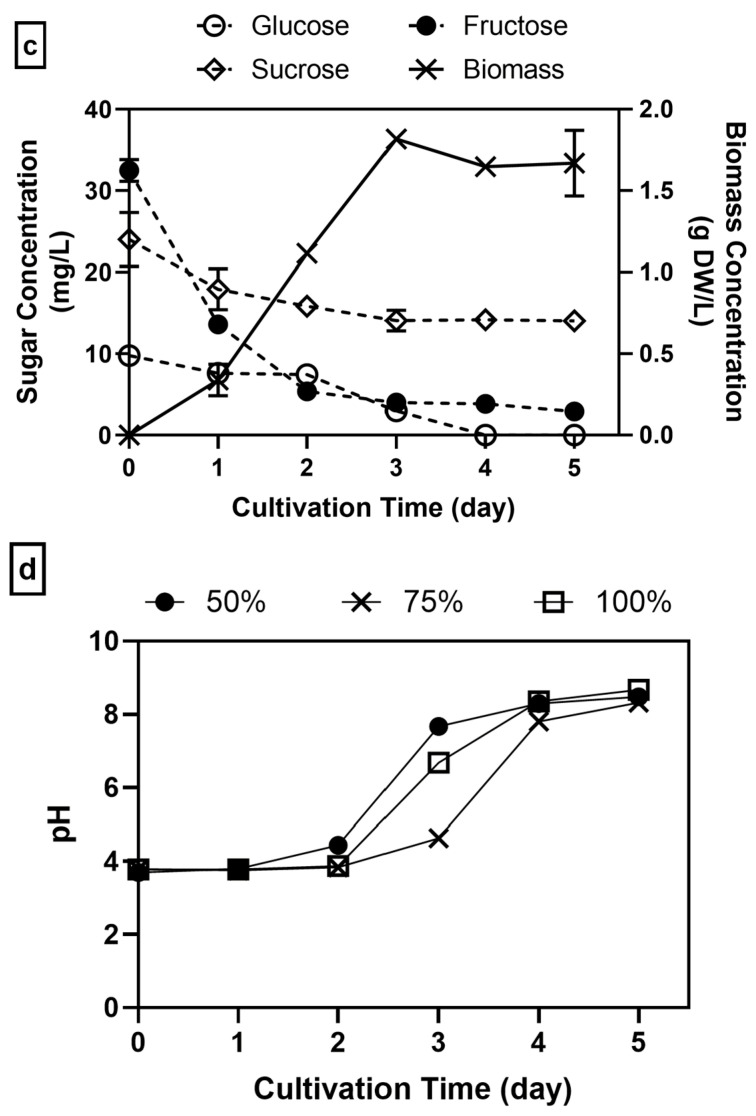
Sugar consumption and biomass production of *A. oryzae* grown in media containing (**a**) 50% soy whey, (**b**) 75% soy whey, and (**c**) 100% soy whey (**d**) and pH trend during 5-day cultivation in 50%, 75%, and 100% soy whey (2% inoculum, 30 °C, 100 rpm).

Figure 2 shows that the sugar concentration increased as the substrate became less diluted. The increase in sugar concentration in the 100% soy whey led to a significant rise in biomass production by 93–173%, from 0.666–0.942 g DW/L to 1.817 g DW/L, compared to 50% and 75% substrate media (*p* < 0.0001). In our previous study, 50% soy whey produced slightly higher biomass (0.87 g DW/L) compared to the present study using the same substrate concentration [28]. This difference might be due to the different batches of soy whey used along with the different inoculum sizes (10% *v*/*v*) [28,44]. Additionally, the highest biomass concentration produced in 100% samples was achieved within 3 days of cultivation, in contrast to diluted samples, which took 5 days to peak.

In all cases, glucose and fructose were rapidly consumed within the first day of incubation, resulting in ~70% and ~88.6% decrease by day 3, respectively. In contrast, sucrose exhibited a slower depletion rate, suggesting that it was a less preferred substrate for growth. Notably, in the 75% soy whey, *A. oryzae* appeared to utilize sucrose to sustain biomass growth after glucose and fructose were fully consumed. The rate of sucrose consumption, however, followed an interesting trend in response to soy whey concentration, with the consumption rate decreasing as the soy whey concentration increased. This is even more pronounced in 100% soy whey, where sucrose consumption slowed significantly after the first day of incubation, leaving nearly 14 mg/L of sucrose unconsumed by the end of incubation (Figure 2c). During this time, biomass growth also plateaued, suggesting that factors other than sugar availability may have limited further biomass production. Interestingly, this cessation of growth coincided with a significant increase in pH after day 3 (from 3.78 to 6.68) (Figure 2d). A similar trend was also observed in the 50% and 75% soy whey, where biomass production also halted as the pH increased sharply to ~7.74. The pH increase could correspond to other metabolic activities, including the breakdown of proteins to ammonia or enzyme production [45,46]. These observations suggest that the alkaline pH may have been a crucial factor limiting biomass production, even in the 100% soy whey [45,47]. Similar inhibition under alkaline conditions was reported by Uwineza et al. [36], where *A. oryzae* was no longer able to consume acetic acid as the sole carbon source when the pH was raised to 8.

This experiment showed that 100% soy whey media gave the highest biomass concentration; therefore, subsequent experiments were carried out with this concentration. Furthermore, the cultivation was carried out for 5 days in this experiment and 7 days (with a 50% soy whey concentration) in our previous study [28]. Indeed, the incubation time for *A. oryzae* can vary from 18 to 144 h, depending on the substrate used, but typically ends when growth starts to plateau (Figure 2d) [24,32,43,48]. As very little to no metabolic activity could be observed after 3 days of incubation, the incubation time for the following experiments was reduced to 3 days accordingly.

#### 3.1.2. Effect of Initial pH

The initial pH of the media is one of the critical factors influencing microbial growth [36,49]. *A. oryzae* is known to grow optimally within the pH range of 5–6 [49], although in some cases, the optimum pH for *A. oryzae* could vary depending on the strain and composition of the substrate [43,50]. While our previous study observed biomass growth in soy whey media with an initial pH of ~3.7, this acidic condition may not have supported optimal growth, potentially limiting biomass production. In the present study, the initial pH of 100% soy whey was adjusted to 4, 5, and 6 prior to inoculation, and the effect of these pH conditions on biomass production was evaluated.

**Figure 3:**
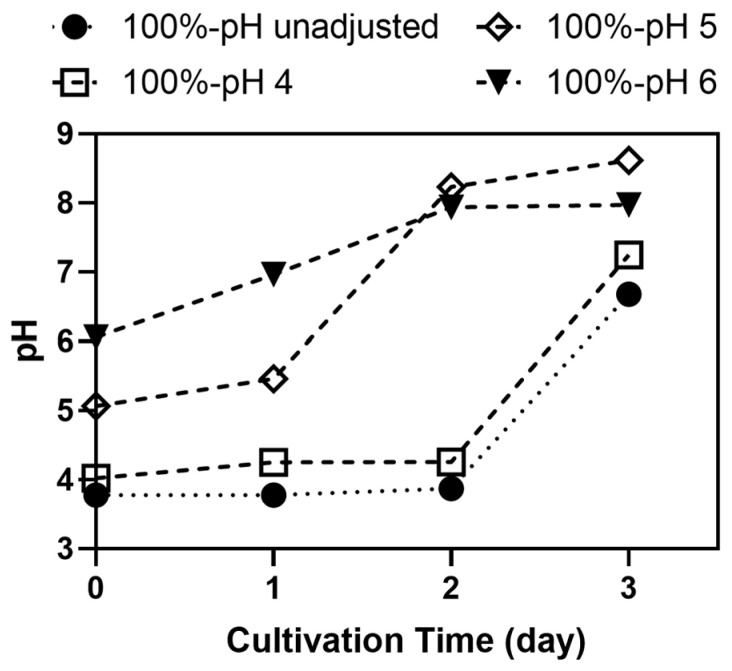
The pH trend of *A. oryzae* during cultivation in 100% soy whey media (2% inoculum, 100 rpm, 30 °C) with adjusted pH (4, 5, and 6) in contrast to samples with unadjusted pH.

As shown in Figure 3, an increase in pH was observed in all samples, consistent with our previous finding [28]. A pH increase during *A. oryzae* cultivation was also reported by Uwineza et al. [36] in the synthetic volatile fatty acid medium due to acid consumption by the fungus. This phenomenon may also have occurred in soy whey media, which indicates the initial presence of acids that were subsequently consumed and resulted in a pH increase. However, the pH increase was delayed in samples with lower initial pH. Samples with an initial pH of 4 followed a similar trend to pH-unadjusted samples (initial pH 3.7), where the pH remained relatively stable for the first 2 days of incubation before rising sharply to ~6.6. In samples with an initial pH of 5, the pH remained relatively constant for the first day, then increased significantly to approximately 8.23 by day 2, followed by a slight increase to ~8.62 on day 3. In contrast, samples with an initial pH of 6 showed a steady increase over the first 2 days, reaching ~7.93, where it remained constant for the rest of the incubation period.

**Figure 4:**
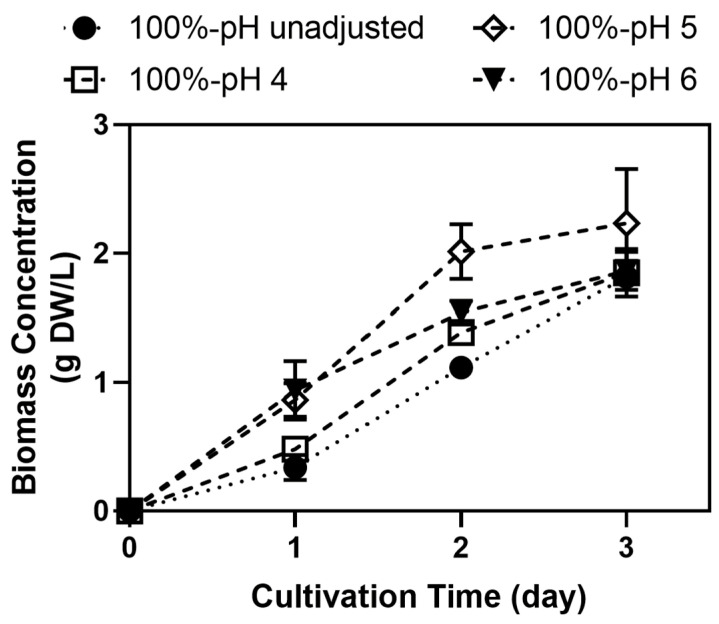
Biomass concentration of *A. oryzae* during 3-day cultivation in soy whey media (100%, 2% inoculum, 30 °C, 100 rpm) with varying initial pH (unadjusted, 4, 5, and 6).

As shown in Figure 4, the initial pH had a clear impact on biomass growth, with an initial pH of 5 resulting in the highest final biomass concentration (2.24 g DW/L). Although the final biomass concentrations between pH-unadjusted and adjusted samples were not significantly different (*p* = 0.1998), adjusting the pH to 5 and 6 was shown to accelerate biomass growth during the early phase. In contrast, growth in samples with unadjusted pH (~3.7) and those adjusted to pH 4 was significantly delayed during the first day of incubation, resulting in a final biomass concentration of 1.82–1.85 g DW/L. This delay could be attributed to the fact that the pH of these samples remained too acidic (below 4) during this period, which is suboptimal for *A. oryzae* growth (Figure 3). In samples with an initial pH of 5, the pH remained stable around this level during the first day of incubation (Figure 3), which likely provided favorable conditions for rapid growth, enabling biomass to peak after only 2 days. In contrast, while samples with an initial pH of 6 exhibited a similar growth rate to those with an initial pH of 5 during the first day, growth slowed down afterward, resulting in a significantly lower final biomass concentration compared to pH 5 samples (1.87 g DW/L).

Tofu production can occasionally involve acids for coagulation, causing the soy whey to possess a low pH [30,41]. Uwineza et al. [36] observed that *A. oryzae* was more capable of utilizing acids at a higher pH. This may have caused the earlier pH increase in pH 5 and 6 samples (Figure 4). Alternatively, *A. oryzae* utilized not only sucrose, fructose, and glucose but also the other oligosaccharides in soy whey media, particularly raffinose and stachyose [30,51]. The higher initial pH potentially allowed more enzyme production and activity, allowing *A. oryzae* to break down more complex sugars and fructose production [30,52,53]. Nevertheless, due to the higher biomass concentration, the subsequent experiment was carried out in soy whey media with pH adjusted to 5.

#### 3.1.3. Effect of Nitrogen and/or Mineral Supplementation

Nitrogen sources and minerals are essential for the growth and metabolic activity of microbes, including *A. oryzae*. In fact, previous studies have reported a positive effect of supplementing various substrates with nitrogen sources and minerals on *A. oryzae* biomass production [36,39,47]. With this in mind, this experiment investigated the effect of adding a nitrogen source (8 g/L ammonium sulfate) and/or minerals (0.75 g/L MgSO_4_·7H_2_O, 1 g/L CaCl_2_·H_2_O, and 3.5 g/L KH_2_PO_4_). Ammonium sulfate provides ammonium, which is a readily assimilable nitrogen source that can be directly incorporated into amino acids via transamination reactions [36,54]. Indeed, ammonium sulfate is commonly used as a cheaper alternative to complex nitrogen sources to promote the growth and enzymatic activity of *A. oryzae* [47,50,55]. Additionally, the combined use of these mineral salts has been shown to significantly enhance biomass yield and substrate consumption rates by *A. oryzae* in synthetic volatile fatty acid media, compared to using each salt individually [36]. Lastly, the presence of both minerals and nitrogen was also able to increase biomass production in other studies utilizing various wastes, including spent sulfite liquor, volatile fatty acids, and fish processing wastewater [25,26,36].

**Figure 5:**
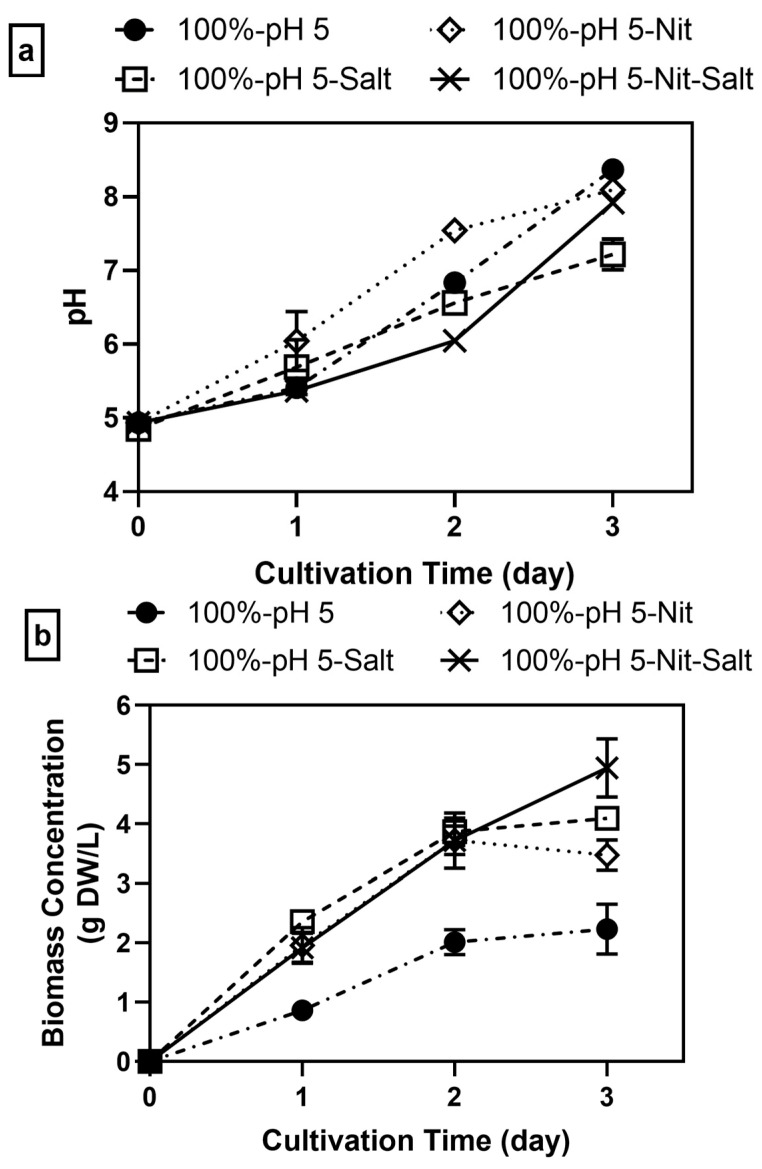
(**a**) pH trend and (**b**) dry biomass concentration of *A. oryzae* cultivated from 100% soy whey media (2% inoculum, 30 °C, 100 rpm, pH 5) with the addition of nitrogen (nit), minerals (salt), or both nitrogen and minerals (nit-salt).

Overall, regardless of nitrogen and/or mineral supplementation, all samples experienced a similar increase in pH, resulting in a final pH of 7.2 to 8, but at different rates (Figure 5a). Nitrogen-supplemented samples had reached a pH of 7.54 by day 2, and afterward, stagnation in both biomass growth and pH increase was observed (Figure 5a,b). In comparison, the pH increase in mineral-supplemented samples was much slower, ~6.56 by day 2 and 7.22 by day 3, which allowed more biomass to be produced. The slowest pH increase was observed in samples supplemented with both nitrogen and minerals, which only reached ~6 by day 2, which extended the growth of *A. oryzae* until the end of cultivation. Minerals also not only promote enzyme production but may provide buffering capacity, which slows down pH increase [55,56].

Furthermore, supplementation with nitrogen, minerals, or both in pH-adjusted 100% soy whey had a massive impact on biomass production by *A. oryzae* (Figure 5b). Nitrogen addition peaked earlier by day 2 at 3.72 g DW/L of biomass concentration, 90% higher compared to unsupplemented samples at their peak. Meanwhile, mineral-supplemented samples produced 4.09 g DW/L of biomass by day 3, 82.5% higher compared to unsupplemented samples. When both minerals and nitrogen were added to the pH-adjusted media, the final biomass concentration increased again to 4.94 g DW/L, 21 and 32.8% higher compared to samples with only minerals or nitrogen added, respectively.

**Figure 6:**
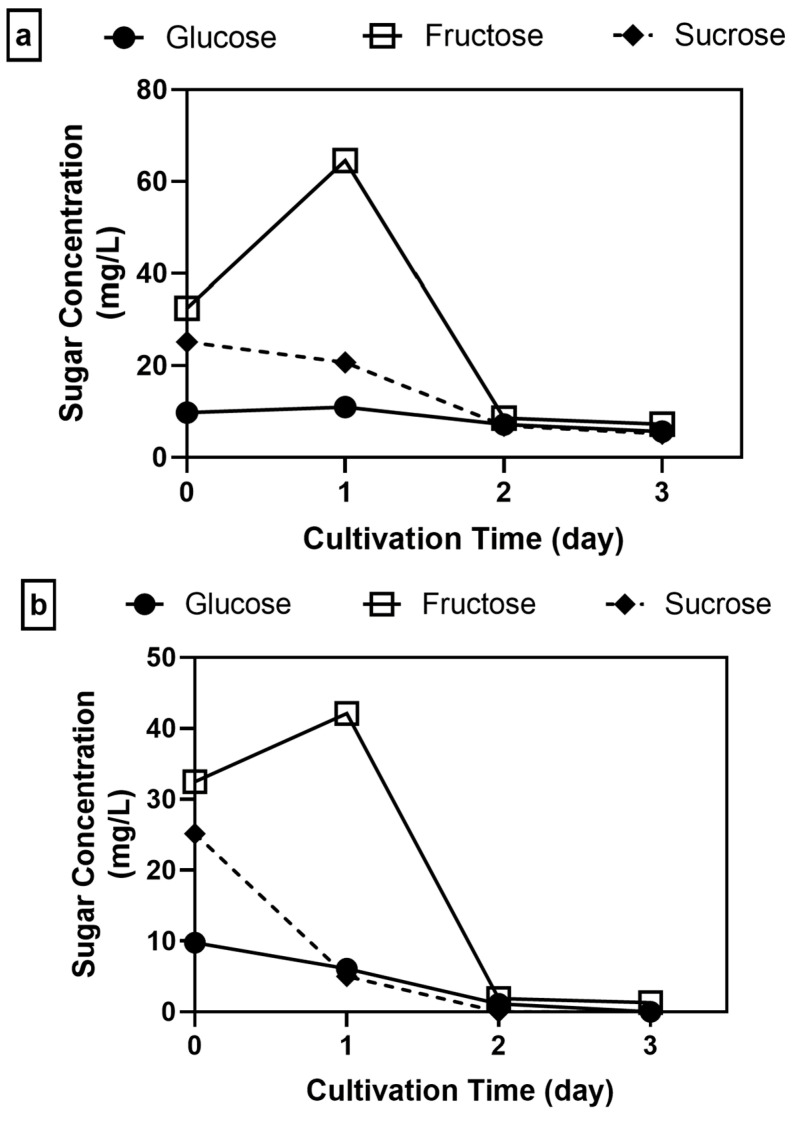

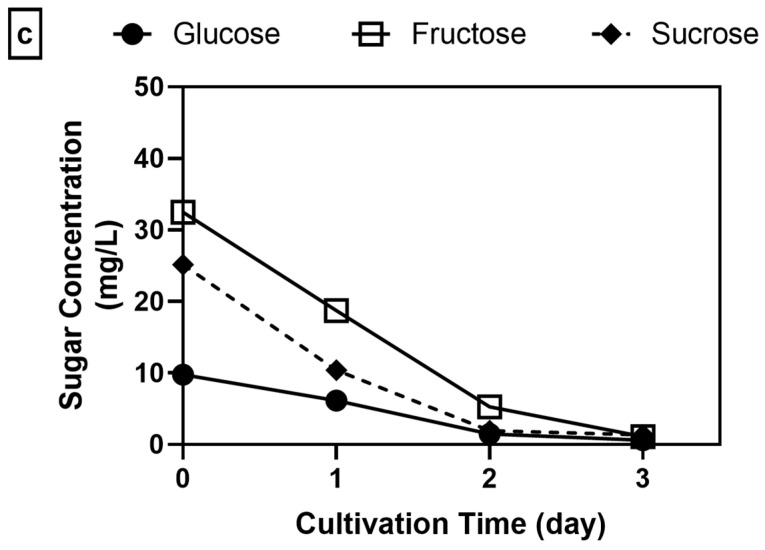
Concentrations of glucose, fructose, and sucrose during cultivation of *A. oryzae* in 100% soy whey with initial pH adjusted to 5 (**a**) unsupplemented or (**b**) supplemented with nitrogen or (**c**) minerals (2% inoculum, 30 °C, 100 rpm).

The sugar consumption was also analyzed to further investigate whether the improved biomass production in pH-adjusted and supplemented media was due to enhanced fungal ability to fully utilize sugars. In comparison to pH-unadjusted and unsupplemented samples (Figure 2c), samples with initial pH adjusted to 5 without supplementation showed an initial ~99% increase in fructose, followed by a ~74% total sugar consumption by the end of cultivation (Figure 6a). Other pH-adjusted samples with nitrogen supplementation also showed a slight initial increase in fructose by 29.7% (Figure 6b), while a consistent decline in sugar concentrations was observed in minerals-supplemented samples from the start (Figure 6c). Interestingly, either nitrogen or mineral supplementation alone increased total sugar consumption to 95–98%, respectively, by the end of cultivation.

These findings indicate that higher biomass production or productivity in supplemented samples was associated with higher sugar consumption. The optimal pH for enzyme production by *A. oryzae* has been observed to be around 5, which may explain the increase in fructose production from the breakdown of larger sugars, such as sucrose [57,58]. Meanwhile, the addition of nitrogen or minerals improved sugar consumption, implying that supplementation enabled *A. oryzae* to be more efficient in substrate utilization, which consequently allowed more biomass to be produced (Figure 5b). A similar finding was also observed by Kövilein et al. [47], where biomass production increased from acetate-based media when ammonium sulfate was added along with Hutner’s mineral solution.

#### 3.1.4. Biomass Yield After Optimization of Cultivation Conditions

**Figure 7:**
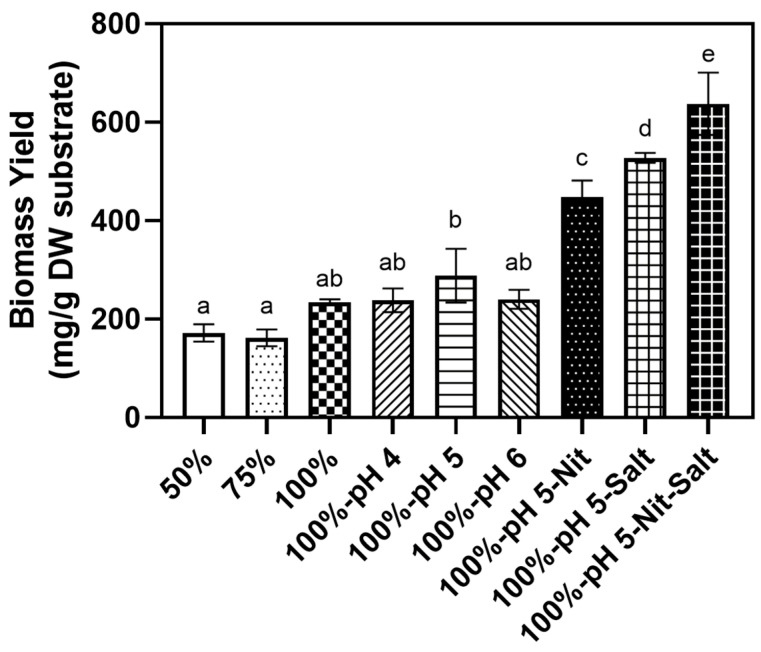
Final biomass yield of *A. oryzae* from different soy whey media conditions after 3 days of incubation (2% inoculum, 30 °C, 100 rpm). Different letters (a,b,c,d,e) indicate significantly different values (*p* < 0.05).

Finally, the biomass yield (mg/DW g substrate) of *A. oryzae* obtained from soy whey media was calculated by dividing the obtained dried biomass (mg) by the amount of dried substrate (g). As shown in Figure 7, the biomass yield of 100% pH-unadjusted, unsupplemented soy whey obtained after 3 days of cultivation (234 mg/g DW substrate) was 1.37-fold higher compared to 50% samples after 5 days of cultivation (*p* < 0.05). When the initial pH was adjusted to 5 in 100% soy whey samples, the biomass yield increased to 288.2 mg/g DW substrate (*p* = 0.3012). The addition of nitrogen or minerals to initial pH 5 samples further increased the biomass yield to 448.14 and 538.44 mg/g DW substrate, respectively (*p* < 0.005). When both minerals and nitrogen were added to pH 5 media, the biomass yield further increased to 637.68 mg/g DW substrate, 42.3% and 20.9% compared to when only nitrogen or minerals were added, respectively (*p* < 0.05). Furthermore, the optimized media achieved 56% higher biomass yield compared to the biomass yield obtained from our previous study [28]. The obtained biomass yield of *A. oryzae* in this study is also higher compared to other substrates reported in previous studies, such as pea-processing by-product, volatile fatty acids, and pomegranate peel [24,36,39]. In addition, the obtained biomass yield is also higher compared to other edible fungi, including triple that of *Mucor indicus* in acid hydrolysate [59] and double that of *N. intermedia* from semi-synthetic media [60].

### 3.2. Protein Content and Yield of A. oryzae Biomass After Optimization of Cultivation Conditions

In our previous study, we achieved a 53% *w*/*w* protein content in mycelial biomass grown on 50% soy whey media (initial pH 3.7) after 5 days of cultivation with 10% inoculum [28]. Building on these findings, the current study aims to further enhance biomass production and ultimately increase protein yields in soy whey substrate.

**Figure 8:**
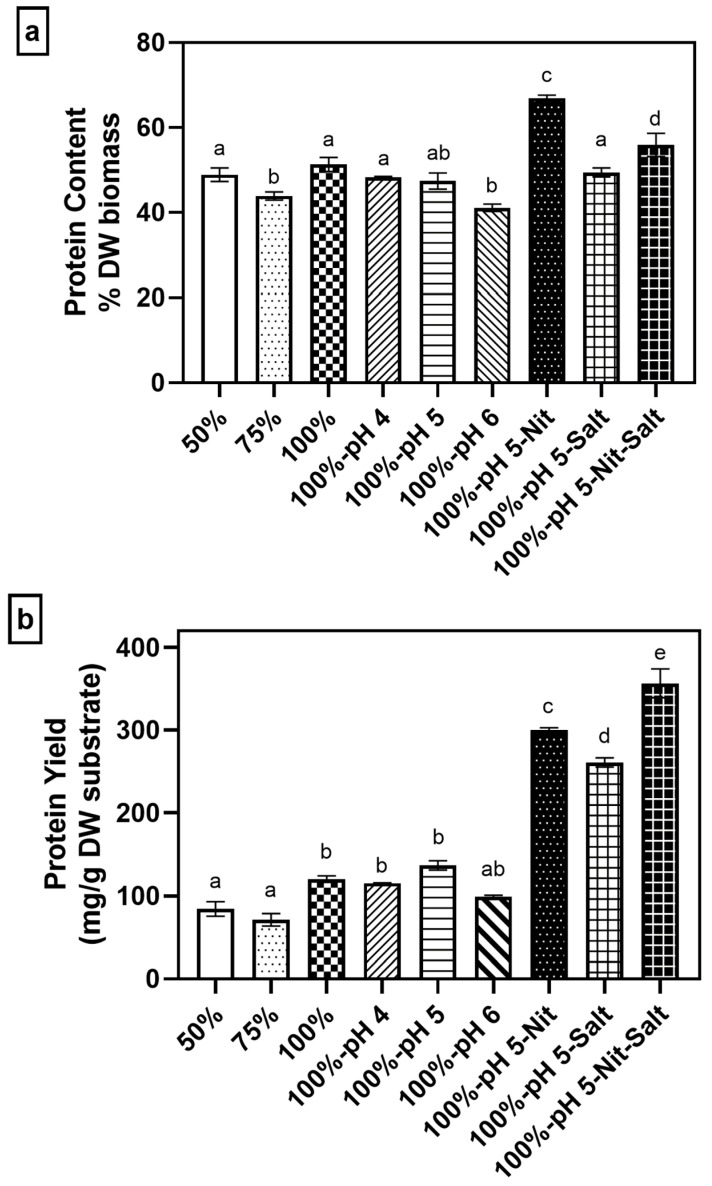
(**a**) Protein content and (**b**) yield of biomass from *A. oryzae* per dried substrate after 3 days of cultivation in soy whey media (2% inoculum, 30 °C, 100 rpm). Different letters (a,b,c,d,e) signify significantly different values among samples.

As shown in Figure 8a, the protein content of *A. oryzae* dried biomass ranged from 41.08% to 66.96% (*w*/*w*), depending on the cultivation conditions used. The highest protein content (66.96% *w*/*w*, *p* < 0.0001) was achieved with nitrogen supplementation alone. The protein content obtained in this study is higher [43,61] or similar to other studies from other wastes [26,62] and commercially available mycoprotein (~45–54% *w*/*w*) [17]. Interestingly, when nitrogen supplementation was combined with minerals, the protein content decreased to 55.93% *w*/*w* (*p* < 0.0001). However, this combined supplementation resulted in the highest total protein yield (356 mg protein/g DW substrate, *p* < 0.0001; Figure 8b), surpassing the yields from nitrogen-only supplementation (300.09 mg protein/g DW substrate) and minerals-only supplementation (260.9 mg protein/g DW substrate). This protein yield is higher compared to our previous study (216 mg/g DW substrate) [28] and is comparable to other studies, including pomegranate and pea-processing by-products (198.6–260 mg/g DW substrate) [24,39].

These findings highlight a trade-off between biomass production and protein accumulation depending on the supplementation strategy used. Nitrogen supplementation alone promotes higher protein content per unit biomass but results in lower overall biomass production, while combining nitrogen and minerals shifts this balance toward increased biomass production at the expense of lower protein content per unit biomass. Nitrogen supplementation has been shown to improve fungal biomass protein content in other studies as well [39,43]. In contrast, mineral addition is found to promote higher biomass production. As an example, calcium is known to promote conidiation in fungi [63,64]. In fact, the biomass obtained from nitrogen and mineral-only samples resulted in the largest and smallest pellet sizes, respectively. Many studies have shown that the substrate and supplementation can impact how the fungal cells aggregate, which in turn results in different pellet sizes [65,66]. The larger pellets observed in nitrogen-supplemented cultures suggest a growth pattern focused on protein assimilation and cellular differentiation rather than rapid proliferation. Such larger pellets generally have reduced nutrient and oxygen diffusion into their cores, potentially restricting overall biomass accumulation. In contrast, the smaller pellets found in mineral-supplemented samples indicate rapid, dispersed growth with better nutrient and oxygen availability. This condition promotes increased metabolic activity, resulting in enhanced overall biomass production at the expense of protein accumulation [38,63,67].

The use of ammonium sulfate as the sole supplement could be more cost-effective to obtain protein-dense biomass without the addition of other minerals. However, complete supplementation still results in higher biomass yield with a relatively high protein content and is more productive than only nitrogen supplementation. Lastly, further investigations could focus more on the amino acid composition to determine the quality of the obtained mycoprotein and cost analysis to determine if complete supplementation is more cost-efficient [45,68].

### 3.3. COD Removal from Soy Whey by A. oryzae

Soy whey is generated during tofu production and, when discarded, is known for its low pH, high COD, and unpleasant odor; hence, the waste needs to be treated prior to disposal [30,69,70]. There have been several proposed solutions to mitigate this issue, including using the wastewater for anaerobic digestion [71], nutrient recovery [70], alternative substrate for other fermentations [72,73], or even for consumption as a beverage or food product [31,35]. By consuming the sugars and utilizing other nutrients within the substrate, *A. oryzae* has been found to be capable of reducing COD levels in other wastewaters [25,38]. Therefore, this experiment aimed to investigate if *A. oryzae* was also capable of COD reduction in soy whey during biomass cultivation. COD levels in tofu wastewater tend to vary depending on the type of beans used and can vary from 7000 to 14,000 mg/L [70,74], which exceeds the local regulations for wastewater disposal (~300 mg/L) [74]. Indeed, the soy whey used in this study was found to contain a COD level of ~8250 mg/L COD (Table 1). By the end of cultivation, *A. oryzae* was able to reduce COD levels by 37.7% in 100% soy whey without pH adjustment or supplementation, which increased to 59.7% in pH-adjusted media with complete supplementation (*p* = 0.0001). The COD removal achieved by *A. oryzae* is comparable to other methods, such as biofiltration [75,76], and essentially, *A. oryzae* cultivation in soy whey could potentially ease the disposal process. The higher COD removal in optimized media is likely due to higher metabolic activity and increased biomass production (Figure 5 and Figure 6). These findings have also been observed in other studies where supplementation allowed better fungal activity and efficiency in COD removal [62,65,77].

## 4. Conclusions

This study aimed to improve the cultivation conditions via a step-wise OFAT approach to promote the growth of *A. oryzae* in soy whey as a substrate for high mycoprotein production. Indeed, the optimal soy whey concentration was determined to be 100% with an incubation period of 3 days. Further adjustment of the pH of the soy whey, along with the addition of ammonium sulfate and minerals, gave the highest dried biomass concentration (4.94 g DW/L) with a protein concentration of (55.93% *w*/*w*). The selected condition not only increased biomass concentration by 5.68-fold compared to our previous study but also improved the biomass and protein yield per dried substrate (637.68 mg/g and 356 mg/g, respectively). At the same time, cultivation of *A. oryzae* in soy whey media was also able to reduce the COD concentration by 59.7%, showing potential for waste treatment. While the present study improved biomass and protein yield by varying one factor at a time, it did not address possible interactions between parameters. Future research should, therefore, employ multifactorial optimization or response surface methods to identify true combined optima. In addition, further study can be performed on increasing the scale of production while also analyzing the nutritional profile of the mycelial biomass obtained from optimized soy whey media.

## Figures and Tables

**Table 1:** COD removal in soy whey media by *A. oryzae* with and without any pH adjustment and supplementation before and after 3 days of cultivation at 100 rpm at 30 °C. Values with different letters (a,b,c) indicate significant differences (*p* < 0.05).

	COD Concentration (mg/L)	
Sample	Day 0	Day 3
100% soy whey	8400 ± 81.7 ^a^	5233 ± 125 ^b^
100% soy whey + nitrogen + minerals (pH 5)	8100 ± 163 ^a^	3267 ± 419 ^c^

## Data Availability

The original contributions presented in this study are included in the article. Further inquiries can be directed to the corresponding author.

## Abbreviations

The following abbreviations are used in this manuscript:

COD	chemical oxygen demand
DW	dry weight
Nit	nitrogen addition
Salt	minerals addition
